# Testing the Cre-mediated genetic switch for the generation of conditional knock-in mice

**DOI:** 10.1371/journal.pone.0213660

**Published:** 2019-03-13

**Authors:** Mattia Capulli, Rossella Costantini, Stephan Sonntag, Antonio Maurizi, Chiara Paganini, Luca Monti, Antonella Forlino, Doron Shmerling, Anna Teti, Antonio Rossi

**Affiliations:** 1 Department of Biotechnological and Applied Clinical Sciences, University of L'Aquila, L'Aquila, Italy; 2 Department of Molecular Medicine, Unit of Biochemistry, University of Pavia, Pavia, Italy; 3 PolyGene AG, Rümlang, Switzerland; 4 ETH Phenomics Center (EPIC), ETH Zürich, Zürich, Switzerland; 5 Scuola Universitaria Superiore IUSS, Pavia, Italy; Montana State University Bozeman, UNITED STATES

## Abstract

The Cre-mediated genetic switch combines the ability of Cre recombinase to stably invert or excise a DNA fragment depending upon the orientation of flanking mutant loxP sites. In this work, we have tested this strategy *in vivo* with the aim to generate two conditional knock-in mice for missense mutations in the *Impad1* and *Clcn7* genes causing two different skeletal dysplasias. Targeting constructs were generated in which the *Impad1* exon 2 and an inverted exon 2* and the *Clcn7* exon 7 and an inverted exon 7* containing the point mutations were flanked by mutant loxP sites in a head-to-head orientation. When the Cre recombinase is present, the DNA flanked by the mutant loxP sites is expected to be stably inverted leading to the activation of the mutated exon.

The targeting vectors were used to generate heterozygous floxed mice in which inversion of the wild-type with the mutant exon has not occurred yet. To generate knock-in mice, floxed animals were mated to a global Cre-deleter mouse strain for stable inversion and activation of the mutation. Unexpectedly the phenotype of homozygous *Impad1* knock-in animals overlaps with the lethal phenotype described previously in *Impad1* knock-out mice. Similarly, the phenotype of homozygous *Clcn7* floxed mice overlaps with *Clcn7* knock-out mice. Expression studies by qPCR and RT-PCR demonstrated that mutant mRNA underwent abnormal splicing leading to the synthesis of non-functional proteins. Thus, the skeletal phenotypes in both murine strains were not caused by the missense mutations, but by aberrant splicing. Our data demonstrate that the Cre mediated genetic switch strategy should be considered cautiously for the generation of conditional knock-in mice.

## Introduction

In the era of big data the question for geneticists is often whether a mutation identified by next generation sequencing in a particular gene can explain the clinical phenotype of the patient. Indeed a crucial issue in biomedical research is to convert sequence information, -omics, analytical and clinical data into knowledge about gene function.

Skeletal dysplasias represent one of the largest classes of birth defects with 436 different disorders that have been clustered in 42 different groups depending on molecular and/or clinical features. More than 350 disease genes have been identified encoding for proteins involved in a wide spectrum of biological functions in cartilage and bone [1, 2]. To shed light on the molecular mechanisms of skeletal disorders, mechanistic studies using *in vitro* and *in vivo* approaches are necessary to elucidate the role of the disease gene in the disorder.

Cell cultures, or *in vitro* studies, provide the first important system to study human diseases, preserving the physiology of living cells and enabling the manipulation under controlled laboratory condition. Patient-derived cells including fibroblasts, induced pluripotent stem cells, primary chondrocytes, osteoblasts and osteoclasts are widely used to model congenital disorders of cartilage and bone [3]; however, their use does not always mirror the whole body condition.

Due to the complexity of skeletal pathologies *in vivo* models represent a need for the field. Recently, *Danio rerio* (Zebrafish) has become an appealing animal model to study skeletal development as well as to test drug’s efficacy [4–6], nevertheless nowadays mouse models still represent the gold standard for bone disease modeling since they are anatomically and physiologically close to humans. The mouse genome can be manipulated in many different ways in order to generate models that carry the same disease causing mutation detected in patients [7]. Moreover, patient specific mutations might be helpful to identify genotype-phenotype correlations and differences in the effects of particular mutations.

For more than 20 years the most suitable technique to generate animal models has been based on homologous recombination in murine embryonic stem cells to change the expression of an endogenous gene [8]. The gene-targeting technique has many advantages related to the fact that homologous recombination defines the site of integration and the genetic change in a very specific manner. Gene-targeting can be designed to introduce different genetic modifications such as gene deletions (knock-out), point mutations (knock-in), gene insertions in a certain locus or chromosomal rearrangements.

Knock-out mice can be also generated by the gene trap strategy, which is based on the disruption of an endogenous gene function by the random insertion of an intronic gene-trap cassette [9]. More recently, the genome-editing CRISPR/Cas9 technology has offered a new, quick and cheap way to model genetic disorders [10].

Genetic changes in the germ line might be useful to track the gene function, but may also result in severe developmental consequences complicating or precluding the experimental analysis (i.e. because of embryonic lethality in knock-outs). To overcome these limits related to constitutive expression of the targeting construct, conditional mice expressing the gene modification only at a specific developmental stage or in selected cells have been generated. Different conditional systems are available; among them, the tetracycline (tet) regulatory system and Cre/*loxP* or Flp/FRT recombination systems are the most widely used [11, 12]. Cre and Flp recombinases mediate different effects on their DNA target sequences including excision, duplication, integration, inversion and translocation depending on the orientation of their specific recognition sequence, namely *loxP* and FRT, respectively.

However, integration and inversion between wild-type *lox*P sites is inefficient due to activation of a vicious cycle of re-excision through intramolecular recombination. To overcome such limitation the left element/right element (LE/RE) mutant strategy using LE mutant *lox* carrying mutations in the left-inverted repeat region and RE mutant *lox* carrying mutations in the right-inverted repeat region can be used [13, 14]. Recombination between a LE mutant *lox* and a RE mutant *lox* results in the generation of a double mutant *lox* site with mutations in both ends and a wild-type *loxP* site. The double mutant *lox* site is no longer a substrate for Cre recombinase; therefore, the recombination reaction proceeds only in the forward direction.

By combining the ability of Cre recombinase to invert or excise a DNA fragment and the use of wild-type and mutant *loxP* sites, an efficient and reliable Cre-mediated genetic switch has been proposed [15]. Through this strategy expression of a given gene can be turned off, while expression of another one can be simultaneously turned on. This innovative, flexible and powerful approach can be used to easily generate many genetic modifications in a conditional manner [16].

The genetic switch has also been proposed to invert a wild-type exon with a mutated one in order to generate conditional point mutations [14]. In the present work, we have tested this powerful tool to generate two conditional knock-in mice bearing patient specific missense mutations causative of two different skeletal disorders: chondrodysplasia with joint dislocations, gPAPP type and autosomal dominant osteopetrosis type 2 (ADO2).

## Materials and methods

### Preparation of the gene targeting vectors and generation of mutant mice

A vector for the stable inversion mediated by Cre-recombinase was generated by gene synthesis with a combination of *lox71* as LE mutant *loxP* (carrying mutations in the left-inverted repeat region) and *loxKR3* as RE mutant *loxP* (carrying mutations in the right-inverted repeat region) in a head-to-head orientation [13]. Different restriction sites were synthesized upstream, downstream and in between the lox sites, to allow insertion of different cassettes. To test the efficiency of the Cre-mediated irreversible inversion we took advantage of an FRT-flanked *neomycin* resistance cassette cloned between the *lox*-sites using *EcoRV* and *EcoRI*. The resulting vector was transformed in *E*.*coli* expressing Cre-recombinase where efficient recombination was confirmed by inversion *in vivo*.

**Fig 1:**
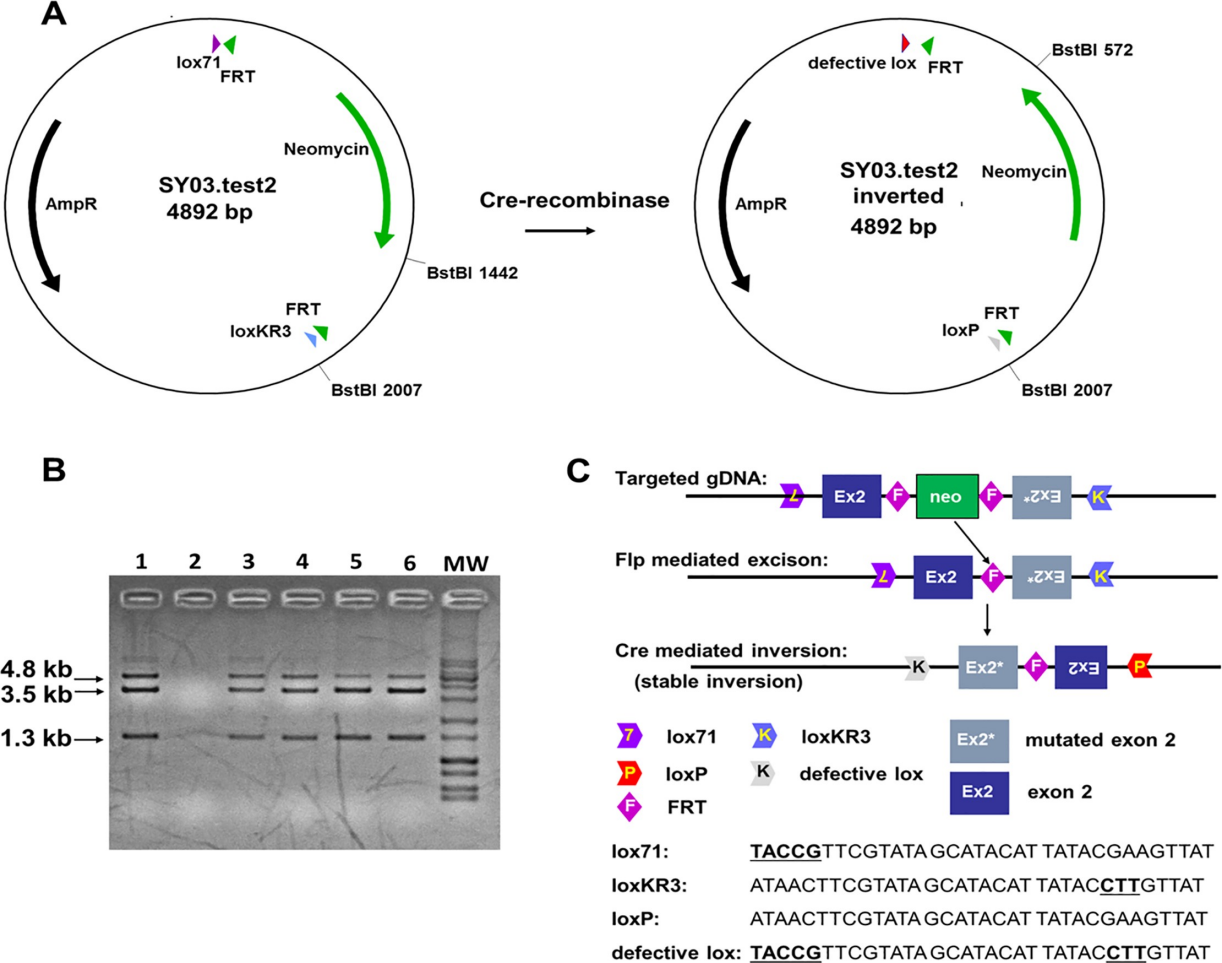
Strategy for conditional knock-in generation via inversion. (A) Scheme of the plasmid used to test recombination and stable inversion with *lox71* and *loxKR3*. Magenta arrowhead: *lox71*; light blue arrowhead: *loxKR3*; grey arrowhead: *loxP*; red arrowhead: double mutant *lox*; green arrowhead: *FRT* site. (B) Restriction fragment analysis with *BstBI* of the plasmid (4.8 kb) after transformation in Cre-expressing *E*.c*oli*. The correctly inverted plasmid results in restriction fragments of 3.5 kb and 1.3 kb. Lanes 1, 3–6: positive clones; lane 2: blank (water); MW: DNA molecular weight marker. (C) Two versions of the targeted exon (wild-type and mutated) are inserted in head-to-head orientation between a *loxKR3* and a *lox71* site. The two versions of the exon are separated by an FRT-flanked *neomycin* cassette. After targeting the *neomycin* cassette is deleted via Flp-mediated recombination *in vivo*. The mutation is activated by stable inversion of the two exons mediated by Cre-recombinase. The targeted exon in the *Impad1* conditional knock-in is exon 2, as shown in the figure, while exon 7 is the targeted exon in the *Clcn7* conditional knock-in. The sequences of *lox* sites used in this study are reported and mutated sequences are indicated by bold and are underlined. *Lox71* carries mutations in the left element, while *loxKR3* carries mutations in the right element.

Subsequently, the *lox71*/*loxKR3* vector was used for the assembly of the *Impad1* and *Clcn7* conditional knock-in gene targeting constructs. Briefly, the vectors contained homology arms for the *Impad1* or *Clcn7* genes of about 5 kb and 2.7 kb or 3.7 kb and 2.8 kb, respectively. Between the homologous sequences a duplicated region of the wild-type and mutated exon (exon 2 for *Impad1* and exon 7 for *Clcn7*) were introduced in a head-to-head orientation; exons were flanked by the head-to-head *lox71* and *loxKR3* sites and separated by an FRT-flanked neomycin cassette (Fig 1).

The missense mutation knocked in the *Impad1* gene was a c.726G>A transition (NM_177730.4) leading to substitution of p.Asp175>Asn in exon 2. This mutation corresponds to the p.Asp177>Asn mutation detected in a patient with chondrodysplasia with joint dislocations, gPAPP type [17]. In addition close to the mutation two silent mutations, c.716T>C and c.719C>T respectively, were inserted to generate a *ClaI* restriction site useful for animal genotyping.

The missense mutation knocked in the *Clcn7* gene was a g.14365G>A transition (NM_011930.4) leading to substitution of p.Gly213>Arg. This mutation corresponds to the most common human mutation, p.Gly215>Arg, detected in patients with ADO2 [18].

### Generation of *Impad1* and *Clcn7* targeted embryonic stem cells and mice

The two targeting vectors bearing the *Impad1* or the *Clcn7* mutation were electroporated into 2 × 10^7^ C57Bl/6N-based and 129Ola-based ES cells, respectively. After selection with 200 μg/ml neomycin (G418) for 8 days, about 400 ES cell clones were isolated and analyzed via PCR screening.

In both ES cell lines, positive ES cell clones in which correct homologous recombination has occurred were identified via long range PCR and confirmed by Southern blot analysis. A list of all primers used for PCR analyses and for the synthesis of the Southern blot probe (generated by PCR with primers Neo3-for and Neo4-rev) as well as for animal genotyping are reported in S1 Table.

For *Impad1* mouse generation, two positive ES cell clones were injected into grey C57Bl/6N derived blastocysts. The resulting chimeras were bred to C57Bl/6N-based Flp-deleter mice (B6gr-Tg(ACTFLPe)9205Dym/NPg) and the resulting black offspring was analyzed by PCR. Several positive mice that lost the FRT-flanked *neomycin* cassette via Flp-recombination were identified (here called "floxed" mice, *Impad1*^*Flox/WT*^ mice). Deletion of the *neomycin* cassette was demonstrated by PCR using primers Neo3-for and Neo4-rev (S1 Table); moreover, the presence of the floxed allele was confirmed by the presence of the *lox71* site checked by PCR with primers SY08.20 and SY08.21. *Impad1*^*Flox/WT*^ mice were mated to a Cre mouse strain (B6.FVB-Tg(EIIa-cre)C5379Lmgd) to generate the heterozygous knock-in of the p.Asp175>Asn mutation (*Impad1*^*D175N/WT*^ mouse). Correct Cre-mediated switch was checked taking advantage of the insertion of the *ClaI* restriction site close to the missense mutation. The 480 bp PCR product of the mutant allele obtained using Imp3 and Imp8 primers was digested by *ClaI* in two restriction fragments of 124 bp and 356 bp, respectively.

For *Clcn7* mouse generation, three positive clones were injected into blastocysts from black C57Bl/6N females. The resulting chimeras were bred to C57Bl/6N-based Flp-deleter (B6gr-Tg(ACTFLPe)9205Dym/NPg) mice. The resulting agouti offsprings were then genotyped and several positive mice showing deletion of the *neomycin* resistance cassette were identified (here called "floxed" mice, *Clcn7*^*Flox/WT*^ mice). These were bred further with each other to generate homozygous floxed mice. For animal genotyping the insertion of the *lox71* site, used as marker of the mutant allele, was checked by PCR with primers flanking the insertion site (SY03.11 and SY03.12). Deletion of the *neomycin* cassette was demonstrated by PCR using primers Neo3-for and Neo4-rev (S1 Table).

### Animals

Animals were bred and maintained in community housing (≤5 mice/cage, 22° C) on a 12 h light/dark cycle with free access to water and standard pelleted food. Care and use of mice for this study were in compliance with relevant animal welfare institutional guidelines in agreement with EU Directive 2010/63/EU for animals, the Italian Legislative Decree 4.03.2014, n. 26 and the Swiss animal protection act (TschG). The experimental protocols were approved by the Italian Ministry of Health (Animal protocol n. 844/2017-PR and n. 564/2016-PR). Research staff received appropriate training in animal care. Mice were euthanized by CO_2_ inhalation before the disease status was considered severe. For genetically modified animals, the evaluation of the severity was based on daily monitoring of the mice behavior, including evaluation of mobility and body weight.

Euthanasia was always done before the animals reached the endpoint, *Impad1* knock-in animals died at birth. The *Impad1* knock-out strain, generated by Frederick et al. [19] was provided by “The Jackson Laboratories”, Bar Harbor, Maine, USA.

### Mouse genotyping

Mice were genotyped by PCR using genomic DNA from mouse tail or ear clips. For animal genotyping of the *Impad1* knock-in strain, the mutant allele was detected by digestion with *ClaI* of a PCR product generated with primers Imp3 and Imp8. The 480 bp PCR product of the mutant allele was digested by *ClaI* in two restriction fragments of 124 bp and 356 bp, respectively. Genotyping of the *Impad1* knock-out strain was performed as described previously [19].

For animal genotyping of the *Clcn7* floxed strain SY03.11 and SY03.12 PCR primers were used to genotype homozygous mutant mice from heterozygous and wild-type animals.

### X-rays and image analysis

X-ray analyses were performed using a Faxitron MX-20 cabinet X-ray system (Faxitron Bioptics LLC, USA) and X-ray images were digitalized with a Kodak DirectView Elite CR System (Carestream Health Italia, Italy).

### Micro computed tomography

Femurs from 3 week-old mice were fixed in 4% formaldehyde for 48 hours and then scanned by a μCT SkyScan 1174 (Bruker Italia, Italy). The scan was performed with 6.4 μm resolution at 50 kV. The Skyscan NRecon software was used to reconstruct images with a modified Feldkamp algorithm. Three-dimensional analysis was carried out employing a Marching Cubes type model with a rendered surface [20]. Threshold values were applied for segmenting trabecular bone. Bone trabecular and cortical variables were determined according to Bouxsein et al [21].

### Differential skeletal staining with alcian blue and alizarin red

Skeletal staining of newborn mice were performed with alcian blue and alizarin red [22]. Briefly, pups were skinned, dehydrated in 96% ethanol, defatted in acetone and stained with alcian blue and alizarin red. Muscles were removed with 1% KOH in 20% glycerol and preparations were stored in glycerol. Mice were then photographed using a Leica M165 FC stereomicroscope connected to a Leica DFC425 C digital camera (Leica Microsystems, Italy).

### qPCR and alternative splicing analysis

Skin from newborn mice was homogenized in 1 ml of QIAzol Lysis Reagent (QIAGEN, Italy) and total RNA was extracted according to the manufacturer’s instructions. Reverse transcription of 1 μg RNA in a final volume of 20 μl was performed using the SuperScript IV First-Strand Synthesis System (Invitrogen, Thermo Fisher Scientific, Italy) according to the manufacturer’s instructions.

For expression analyses by RT-qPCR, the QuantiFast Primer Assays with validated primer sets for *Impad1* and *Gapdh* (QIAGEN) were used with the QuantiTect SYBR Green PCR Kit (QIAGEN) according to the manufacturer’s protocol. The primer set of *Impad1* span exon 2 and 3. Each sample was run in triplicate in 96 well plates in three independent experiments with the MX3000P apparatus (Stratagene, USA). The expression of *Impad1* relative to the housekeeping *Gapdh* gene was obtained by ΔΔCt method.

For alternative splicing detection, total RNA was used for RT-PCR analysis using primers in exon 1 and exon 5 of the *Impad1* gene and in exon 6 and 25 of the *Clcn7* gene. PCR products were analyzed by 1.5% agarose gels. For DNA sequencing of the spliced forms of *Impad1*, the PCR products were cloned with the TA cloning kit (Invitrogen) followed by Sanger sequencing.

### HPLC analysis of cartilage glycosaminoglycan sulfation

For cartilage disaccharide analysis, cartilage was obtained from the femoral heads of newborn mice by careful dissection under the dissection microscope and glycosaminoglycans (GAGs) were recovered by papain digestion and cetylpyridinium chloride precipitation as previously described [23]. Purified GAGs were digested with 30 mU of both chondroitinase ABC and chondroitinase ACII (Seikagaku Corp., Japan) in 0.1 M sodium acetate, pH 7.35 at 37°C overnight. Released disaccharides were lyophilized and redissolved in 40 μl of 12.5 mM 2-aminoacridone (Invitrogen) in 85% DMSO/15% acetic acid and incubated for 15 minutes at room temperature in the dark. Then, 40 μl of 1.25 M sodium cyanoborohydride in water was added and the mixture was incubated overnight at 37°C. Disaccharides were fractioned with a ProntoSIL HPLC column (4.6 mm × 200 mm, Bischoff Chromatography, Germany) using a linear gradient (0–42% solvent B in 42 min); mobile phases were 0.1 M ammonium acetate, pH 7.0, (Solvent A) and methanol (Solvent B). The analysis was carried out at room temperature with 0.7 ml/min flow rate and the elution profile was monitored by a fluorescence detector (2475 Multy λ Fluorescence Detector, Waters, Italy), with excitation and emission wavelengths of 425 and 525 nm, respectively.

### Statistical analysis

Statistical analyses were carried out by the software Prism by GraphPad v7.0 and the type of analysis is stated in the figure legends; all results are presented as mean ± SD.

## Results

### Generation of the Cre mediated switch allele in mice

In order to generate the two conditional knock-in mice, a combination of two partially mutated *lox* sites in the head-to-head orientation was used. This strategy guarantees stable inversion of DNA between the *lox* sites since after Cre recombination a wild-type *loxP* site and a double mutant *lox*, no longer recognized by the enzyme, are generated (Fig 1A). First the combination *lox71* and *loxKR3*, reported previously by Araki et al [13] in a head-to-head orientation was tested for recombination efficiency in a Cre expressing *E*.*coli* strain. Five clones were isolated from each transformation and analyzed via restriction analysis with *BstBI*. All clones showed the expected restriction pattern corresponding to the inverted version of the test vector (Fig 1B).

**Fig 2:**
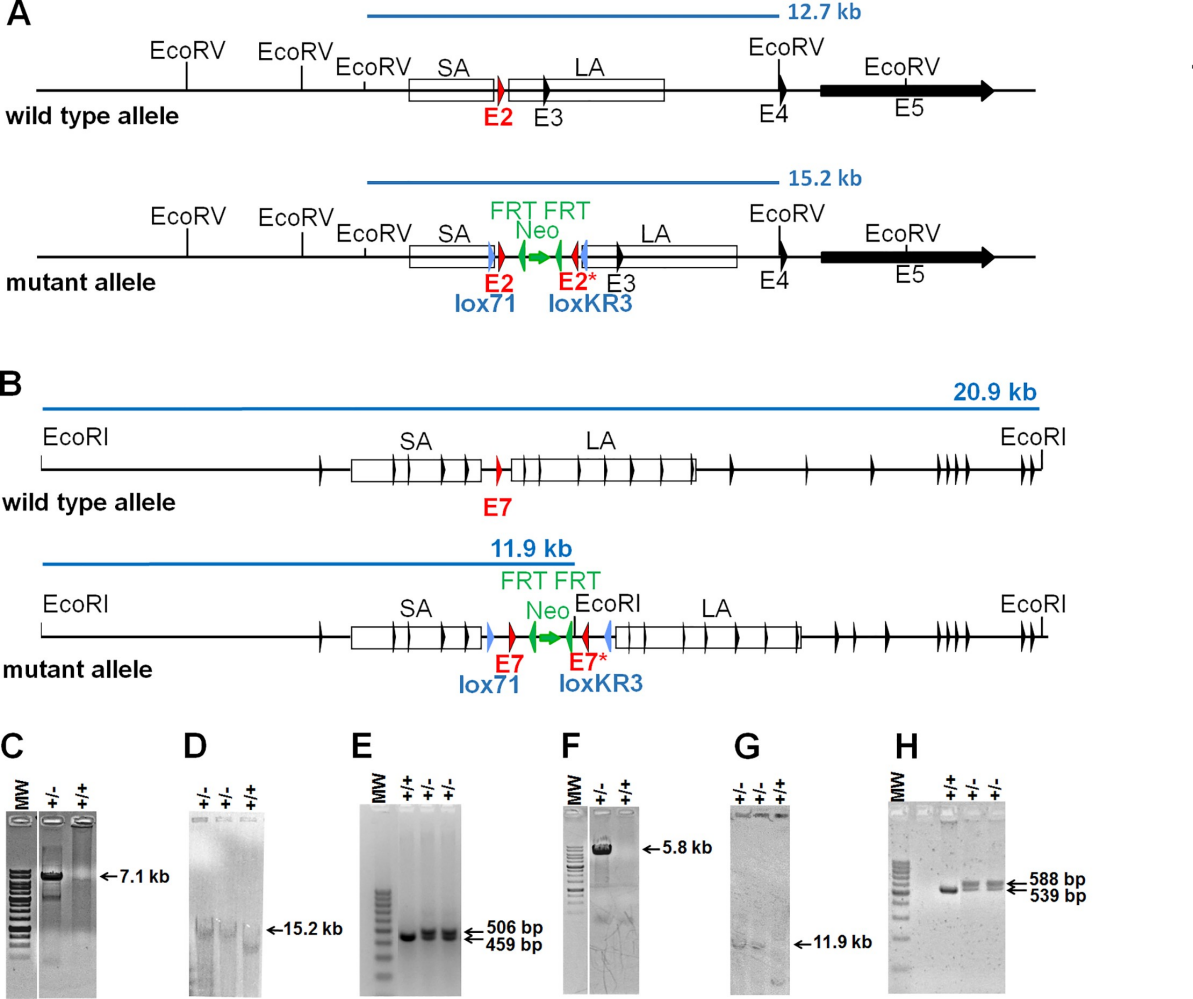
Generation of the *Impad1* and *Clcn7* mouse lines. (A) Schematic drawing of the wild-type and targeted loci for *Impad1*. *EcoRV* restriction sites and fragments for Southern blot analyses are indicated in light blue. (B) Schematic drawings of the wild-type and targeted loci for *Clcn7*. *EcoRI* restriction sites and fragments for Southern blot analyses are indicated in light blue. Exons, indicated by arrowheads, are not numbered to simplify the figure. (C) Long range PCR analysis for correct homologous recombination of ES clones with primers SY08.19 and LRPCRneo1. The expected fragment of 7.1 kb confirms correct homologous recombination in the *Impad1* locus. (D) Southern blot analysis of *Impad1* targeted ES cells after *EcoRV* digestion using a *neomycin* specific probe; the 15.2 kb fragment corresponding to correct homologous recombination is indicated. (E) Genotyping PCR analysis for heterozygous *Impad1* floxed pups using primers SY08.20 and SY08.21 flanking the *lox71* site. The wild-type and floxed allele result in PCR products of 459 bp and 506 bp, respectively. (F) Long range PCR analysis for correct homologous recombination of ES clones with primers SY03.9 and LRPCRneo1. The expected amplicon of 5.8 kb confirms correct homologous recombination in the *Clcn7* locus. (G) Southern blot analysis of *Clcn7* targeted ES cells after *EcoRI* digestion using a *neomycin* specific probe; the 11.9 kb fragment corresponding to correct homologous recombination is indicated. (H) Genotyping PCR analysis for heterozygous *Clcn7* floxed pups using primers SY03.11 and SY03.12 flanking the *lox71* site. The wild-type and floxed allele result in PCR products of 539 bp and 588 bp, respectively. Black arrowhead: exon; green arrowhead: FRT site; light blue arrowhead: *lox71* or *loxKR3* site; red arrowhead: targeted exon 2 in *Impad1* or targeted exon 7 in *Clcn7*; green arrow: *neomycin* cassette; SA: short arm; LA: long arm; E#: exon number; MW: DNA molecular weight marker.

Two gene targeting vectors were generated based on the *lox71*/*loxKR3* construct. The first for an Impad1 mutation (p.Asp175>Asn) and the latter for a mutation in the Clcn7 (p.Gly213>Arg) (Fig 1C and Fig 2A and 2B).

Homologous recombination was performed in C57Bl/6N-based (Impad1) and 129Ola-based (Clcn7) ES cells and confirmed by long range PCR and Southern blot analysis (Fig 2C and 2D for *Impad1* and Fig 2F and 2G for *Clcn7*). Proper targeted ES cell clones were injected into C57Bl/6N blastocysts and the resulting chimeras were bred to FLP-deleter mice for *neomycin* cassette deletion. The heterozygous offsprings, here called “floxed” mice (*Impad1*^*Flox/WT*^ mice and *Clcn7*^*Flox/WT*^ mice, respectively), were then confirmed by the presence of the *lox71* site (Fig 2E and 2H) and the deletion of the *neomycin* cassette based on the absence of a 542 bp fragment after PCR with primers Neo3-for and Neo4-rev.

To generate heterozygous *Impad1* knock-in animals (*Impad1*^*D175N/WT*^), *Impad1*^*Flox/WT*^ mice were mated to a global Cre mouse strain for stable inversion and activation of the mutation.

### Phenotypic characterization of the *Impad1* knock-in mouse

*Impad1*^*D175N/WT*^ mice did not show any phenotypic alteration by X-ray analysis and visual inspection as human carriers of *IMPAD1* mutations and were no further studied.

**Fig 3:**
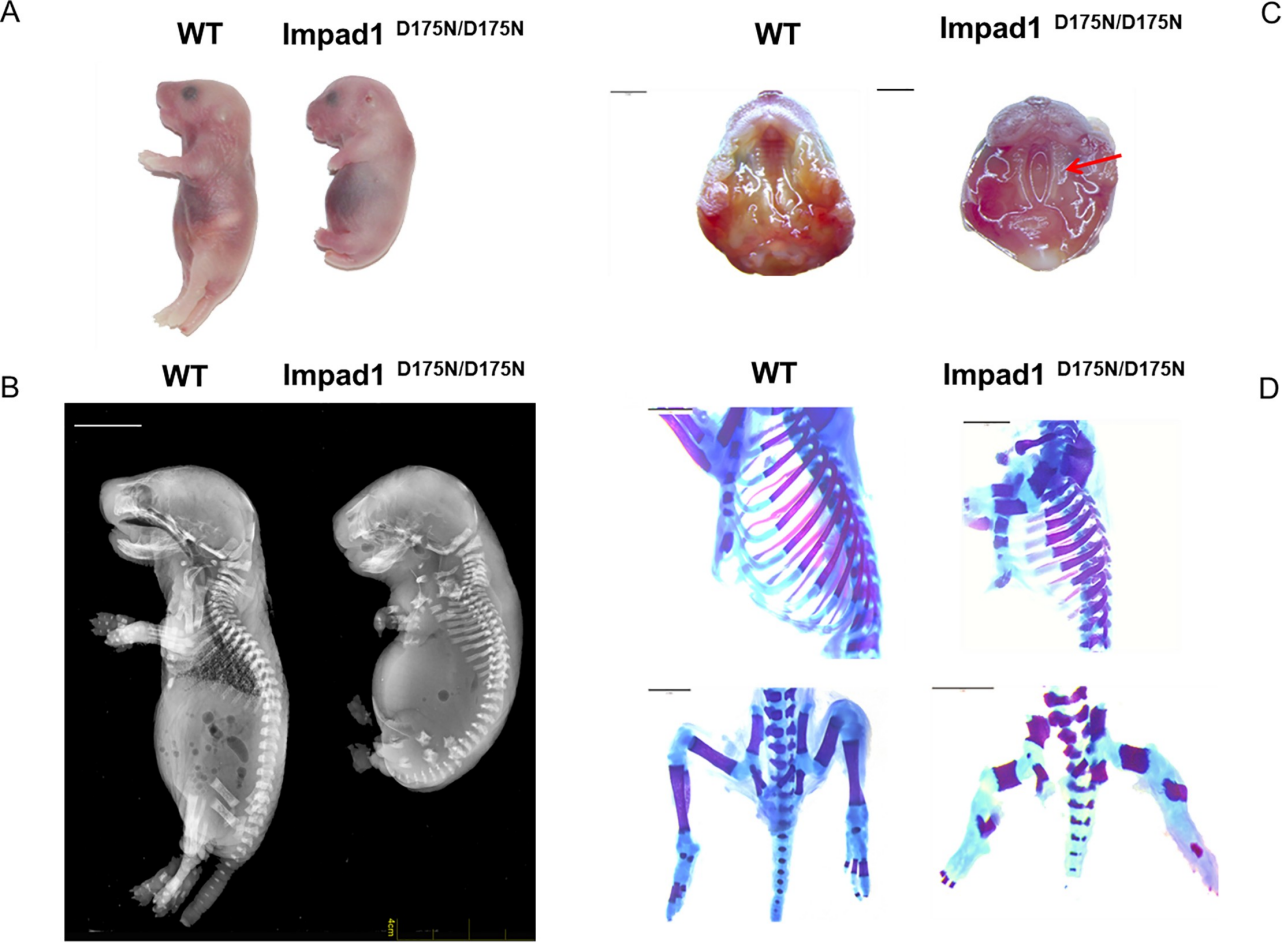
Phenotypic characterization of the *Impad1*^*D175N/D175N*^ mouse. (A) Gross morphology of wild-type and *Impad1*^*D175N/D175N*^ newborn mice. Mutant animals die at birth and are considerably smaller than wild-type littermates demonstrating severe growth retardation. (B) X-rays of wild-type and mutant mice at birth. The mutant shows skeletal defects and growth retardation. (C) In *Impad1*^*D175N/D175N*^ mice cleft palate is observed (arrow). Scale bars: 2 mm. (D) Alcian blue and alizarin red skeletal staining of *Impad1*^*D175N/D175N*^ and wild-type bones at birth; the femur, tibia and fibula are markedly shorter compared to wild-type animals; rib cages of mutant mice display skeletal defects characterized by reduced sternal length and diminished rib spacing. Scale bars: 2 mm.

Homozygous mutant (*Impad1*^*D175N/D175N*^*)* animals died at birth and were smaller compared to wild-type and heterozygous littermates; visual inspection and X-ray analysis demonstrated growth retardation and skeletal defects (Fig 3A and 3B). Mutant animals showed severe hypoplasia of the skeleton; the length of the axial skeleton and of the limbs were reduced. By X-rays and alcian blue and alizarin red skeletal staining, the femur, tibia and fibula were markedly shorter compared to wild-type animals; furthermore, reduced sternal length and diminished rib spacing were evident (Fig 3B and 3D). Moreover, *Impad1*^*D175N/D175N*^ mice showed cleft palate already observed in patients with chondrodysplasia with joint dislocations, gPAPP type (Fig 3C).

Surprisingly, the *Impad1*^*D175N/D175N*^ mouse showed the same peculiar phenotype described previously in the *Impad1* knock-out mouse [19, 24]. Thus, we further investigated the molecular and biochemical basis causing neonatal lethality in our animal model.

**Fig 4:**
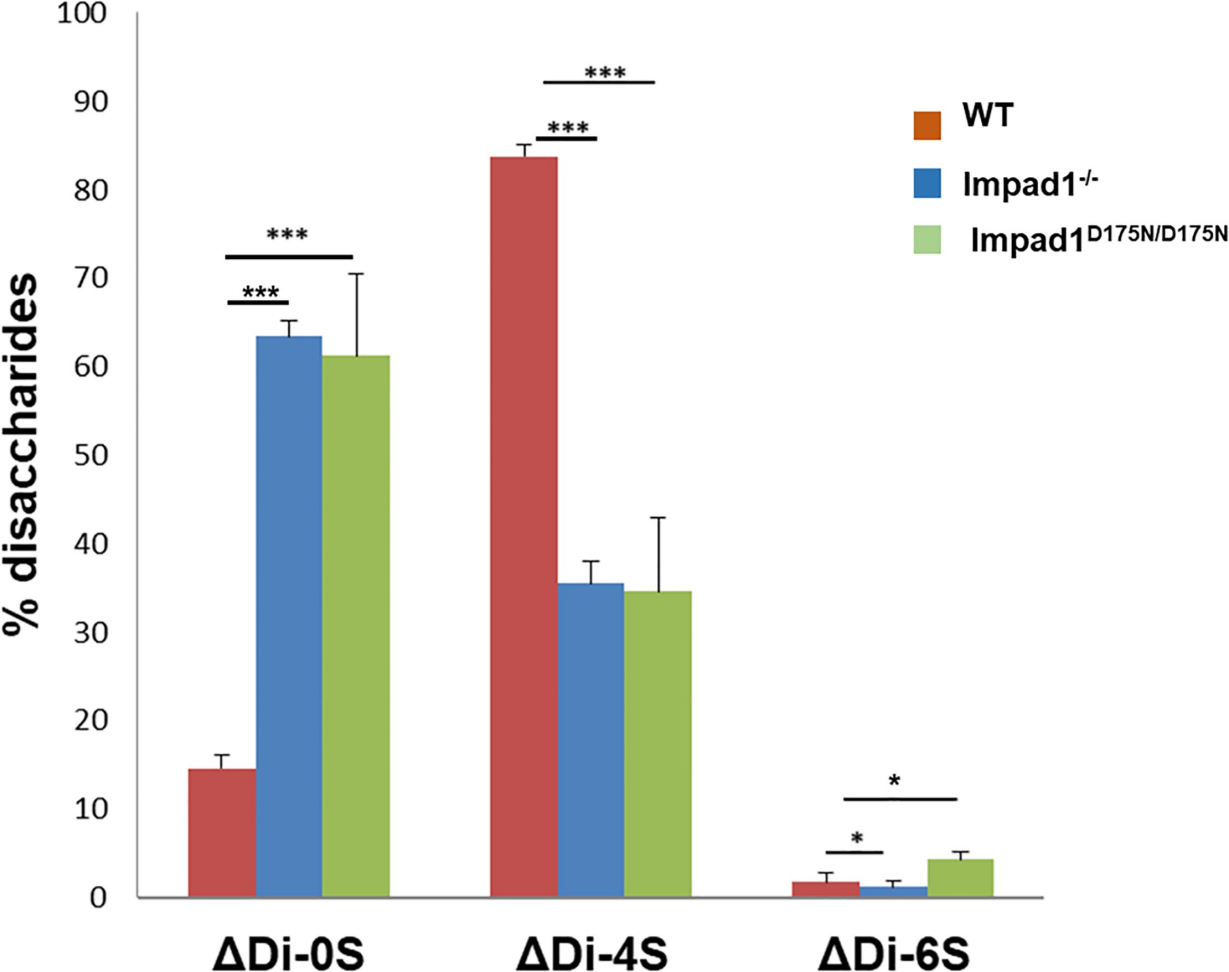
Sulfation of chondroitin sulfate proteoglycans from femoral head cartilage. Sulfation of proteoglycans was determined by HPLC disaccharide analysis after digestion by chondroitinase ABC and ACII of chondroitin sulfate proteoglycans from the femoral head cartilage of wild-type (WT) and *Impad1*^*D175N/D175N*^ mice at birth. In parallel the same analysis was performed also in the *Impad1* knock-out (*Impad1*^*-/-*^) mouse studied by Frederick [19]. The amount of non sulfated disaccharide (ΔDi-0S) relative to the total amount of disaccharides (ΔDi-0S, ΔDi-4S and ΔDi-6S) is significantly increased in mutant mice compared to the wild-types indicating proteoglycan undersulfation. Interestingly, the level of proteoglycan undersulfation in mutants is similar to the Frederick’s knock-out mouse. Three mice per group were used; data are reported as mean ± SD (Student’s t-test, *p<0.05; ***p<0.001).

*Impad1* encodes for a Golgi 3’-phosphoadenosine 5’-phosphate phosphatase crucial for macromolecular sulfation. In whole embryo and in the limbs of the *Impad1* knock-out mouse, severe undersulfation of chondroitin sulfate proteoglycans was demonstrated compared to wild-type mice [19, 24]. For this reason, proteoglycan sulfation was measured by HPLC disaccharide analysis in femoral head cartilage of wild-type and *Impad1*^*D175N/D175N*^ mice and, for comparison, in the Frederick’s *Impad1* knock-out [19]. Glycosaminoglycans were purified by digestion of femoral head cartilage with papain. Then glycosaminoglycans were digested with chondroitinase ABC and ACII and released disaccharides separated by HPLC after derivatization with a fluorescent tag. A dramatic increase in the relative amount of the chondroitin non-sulfated disaccharide (ΔDi-0S) was observed in newborn *Impad1*^*D175N/D175N*^ mice compared to wild-type animals indicating chondroitin sulfate undersulfation (61 ± 9.2% and 14 ± 1.6% ΔDi-0S, respectively; P < 0.001 n = 3). Interestingly, the extent of proteoglycan undersulfation in mutant mice was similar to homozygous *Impad1* knock-out animals (63 ± 1.6% ΔDi-0S, n = 3) (Fig 4).

**Fig 5:**
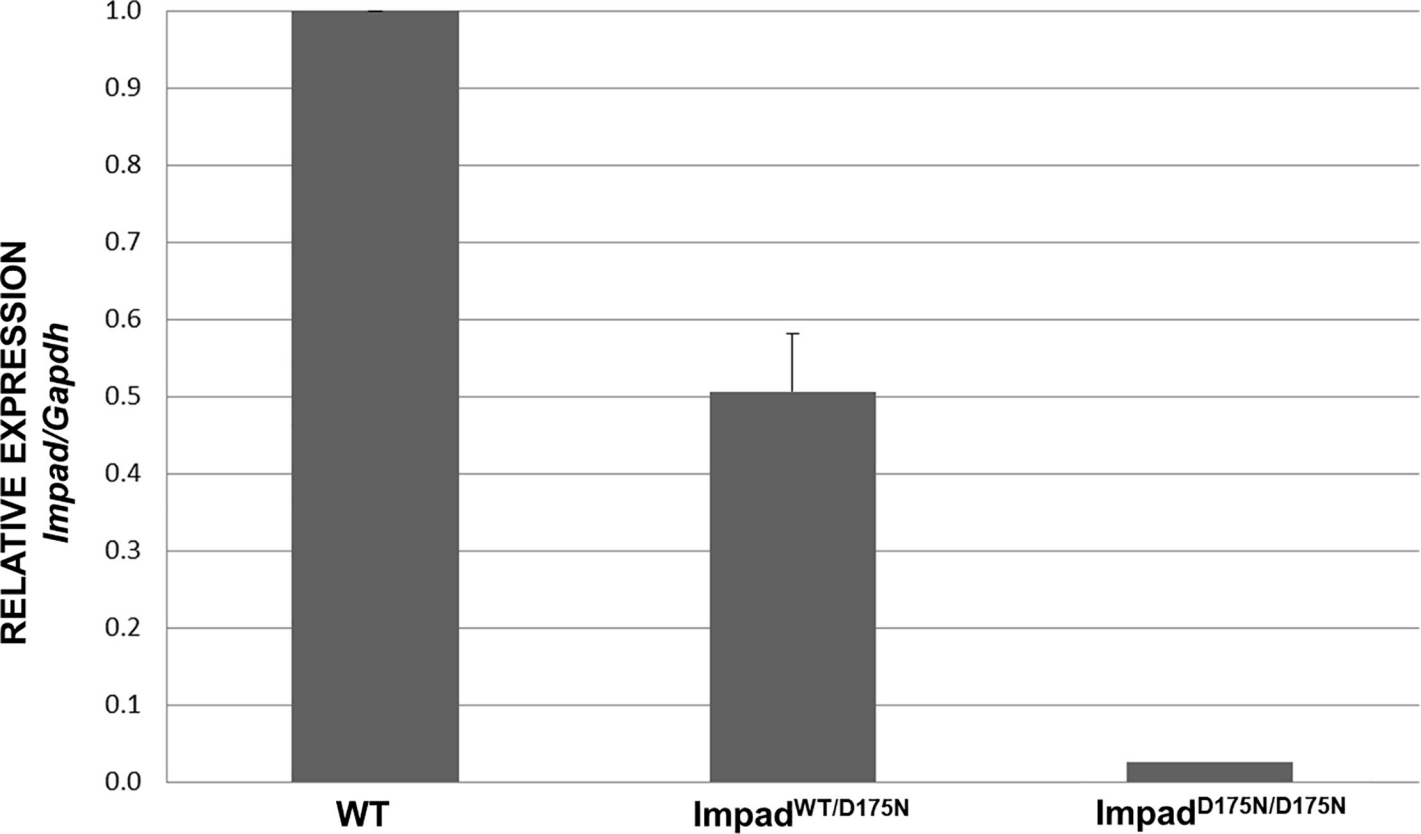
Relative expression analysis of the *Impad1* gene. RT-qPCR on total RNA isolated from skin of wild-type (WT), heterozygous (*Impad1*^*WT/D175N*^) and *Impad1*^*D175N/D175N*^ newborn mice was performed with primers spanning exon 2 and exon 3. Expression of *Impad1* mRNA normalized to *Gapdh* is absent in homozygous mutant animals compared to wild-types. Three mice per genotype were used; each sample was run in triplicate and three different experiments were performed.

Since the clinical and biochemical phenotype of *Impad1*^*D175N/D175N*^ animals overlap with *Impad1* knock-outs, the expression of *Impad1* was measured by qPCR with primers spanning exons 2 and 3. *Impad1* mRNA was barely detectable in *Impad1*^*D175N/D175N*^ mice compared with wild-types; *Impad1* expression level in heterozygous mice was reduced by half (Fig 5).

**Fig 6:**
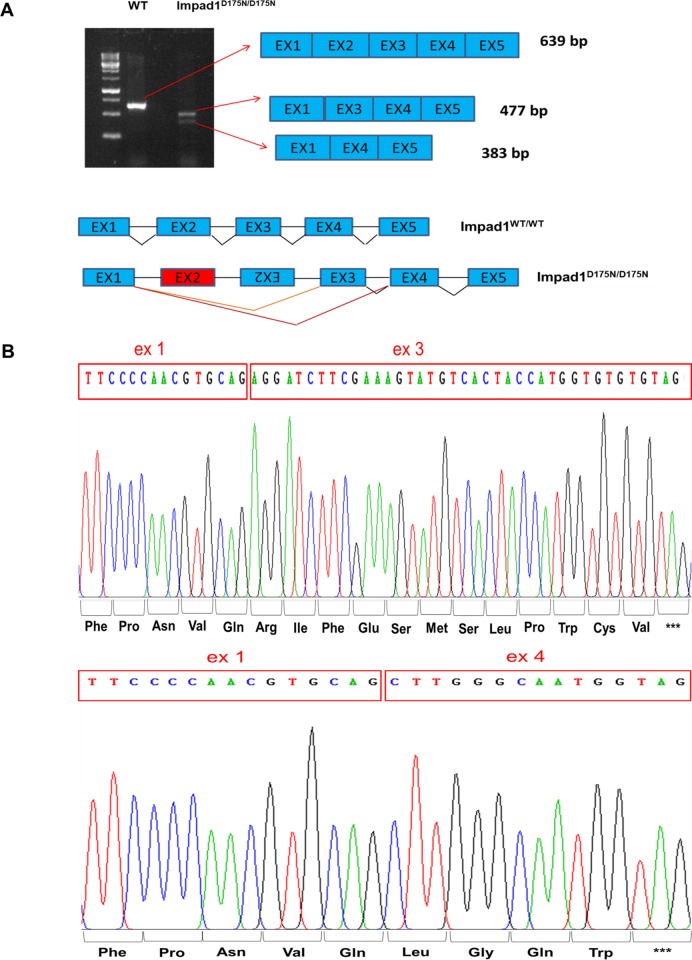
Alternative splicing analysis of *Impad1* mRNA. (A) RT-PCR using primers Imp7 and Imp12 that amplify a region spanning exon 1 to exon 5 was performed from skin total RNA of wild-type and *Impad1*^*D175N/D175N*^ newborn mice and analysed by 1.5% agarose gel. In wild-type animals one band, 639 bp long, corresponding to the correctly spliced *Impad1* transcript including the 5 coding exons is present. This band is not detected in mutant mice, conversely two different bands, 477 bp and 383 bp long, respectively are observed. (B) Sequencing of the two bands demonstrates that the two transcript variants lack exon 2 or both exon 2 and exon 3.

Since in *Impad1*^*D175N/D175N*^ mice no *Impad1* transcript was detected by qPCR, we checked whether the modified *Impad1* locus impairs correct RNA splicing. Thus, we considered potential alternative splicing of *Impad1* exons by RT-PCR using primers Imp7 and Imp12 spanning the coding sequence from exon 1 to 5 (Fig 6). Results showed that in wild-type animals one band, 639 bp long, corresponding to a unique transcript including the 5 coding exons was present. This band was not detected in *Impad1*^*D175N/D175N*^ mice; conversely two different bands, 477 bp and 383 bp long, respectively, were observed (Fig 6A). Sequencing of the two bands demonstrated that the two transcript variants lack exon 2 or exon 2 and exon 3 (Fig 6B) causing, if translated, frameshift mutations leading to premature stop codons p.Ile128ArgfsX13 and p.Ile128LeufsX5, respectively.

Retrospectively we noted that *Impad1*^*Flox/WT*^ cross breeding resulted in an unusual small litters size. The analysis of some pups found dead at birth revealed their homozygous *Impad1*^*Flox/Flox*^ genotype. Furthermore, their X-ray showed the same severe skeletal phenotype observed in *Impad1*^*D175N/D175N*^ mice (S1 Fig).

### Phenotypic characterization of the *Clcn7* floxed mice

**Fig 7:**
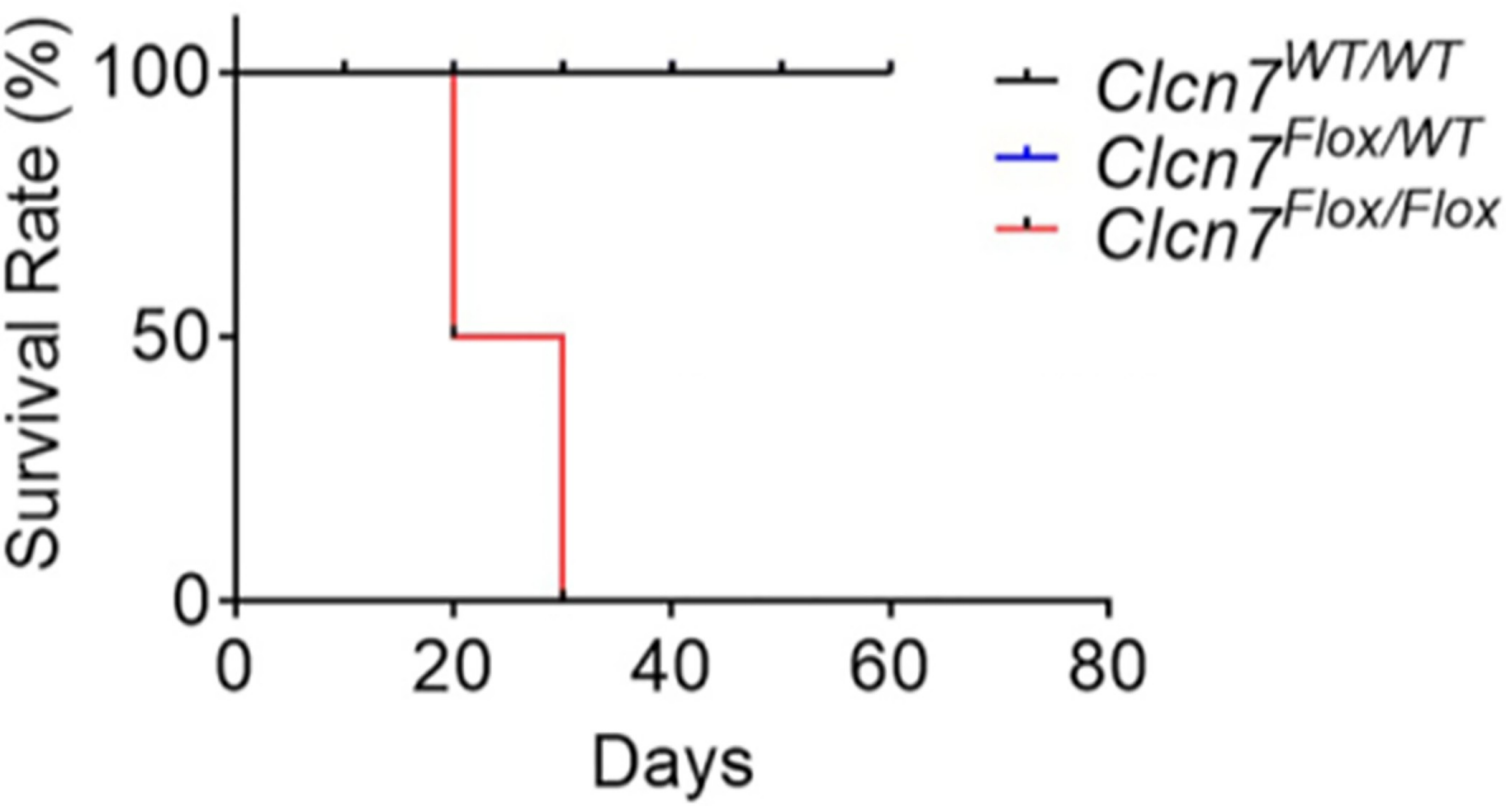
Survival rate of *Clcn7*^*Flox/Flox*^ mice. Survival rates were calculated following *Clcn7*^*WT/WT*^ (black line), *Clcn7*^*Flox/WT*^ (blue line) and *Clcn7*^*Flox/Flox*^ (red line) mice for 60 days. The *Clcn7*^*WT/WT*^ and *Clcn7*^*Flox/WT*^ survival rates overlap, for this reason the blue line is not visible in the graph. Survival rate of *Clcn7*^*Flox/Flox*^ mice is dramatically reduced. Data are the mean ± SD of 5 mice for each group (Mantel-Cox test).

Since the *Impad1*^*D175N/D175N*^ phenotype was not due to the missense mutation, but by altered splicing of the modified *Impad1* allele, and that aberrant splicing was likely responsible for *Impad1*^*Flox/Flox*^ mice unexpected lethal outcome, we checked whether similar modification affected also the *Clcn7*^*Flox/Flox*^ mice. For this reason heterozygous *Clcn7* floxed mice (*Clcn7*^*Flox/WT*^) were mated together to observe the phenotype in homozygous floxed mice (*Clcn7*^*Flox/Flox*^). Since these mice were not mated yet to a Cre expressing murine strain, inversion of the mutated exon, bearing the p.Gly213>Arg mutation, did not occurr yet and thus no phenotype should have been observed. The *Clcn7*^*Flox/Flox*^ mouse presented the phenotypic features of autosomal recessive osteopetrosis. In particular the *Clcn7*^*Flox/Flox*^ displayed a phenotype resembling *Clcn7* knock-out mice [25], with reduced survival (Fig 7) and a normal Mendelian ratio of genotypes at birth. As expected, no significant changes in survival was found in the heterozygous *Clcn7*^*Flox/WT*^ mice (Fig 7).

**Fig 8:**
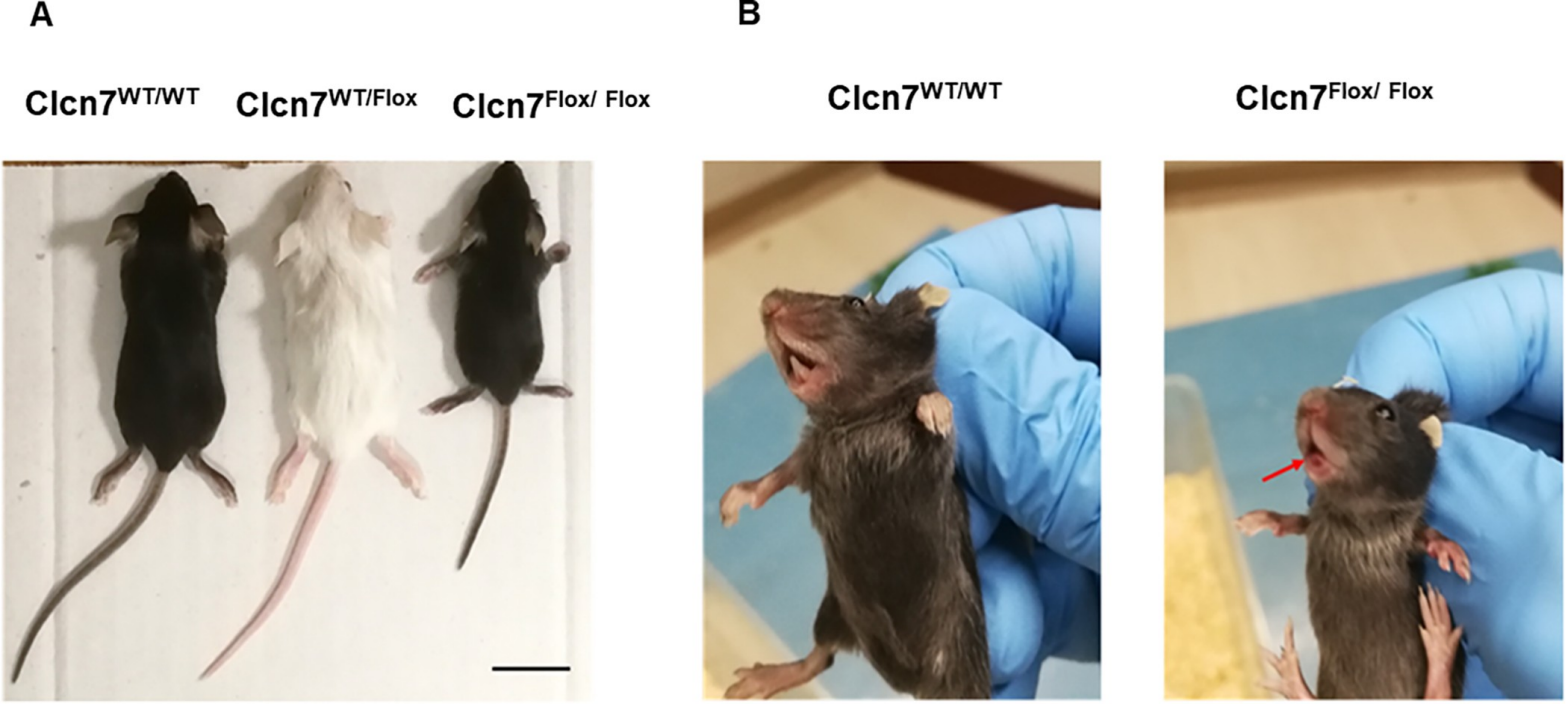
*Clcn7*^Flox/Flox^ mouse phenotype. Twenty-one day old *Clcn7*^WT/WT^, *Clcn7*^Flox/WT^ and *Clcn7*^Flox/Flox^ mice were evaluated for (A) gross appearance and (B) tooth eruption. *Clcn7*^*Flox/Flox*^ mice show reduced skeletal growth and no tooth eruption (the red arrow points to the absence of teeth). Scale bar: 1 cm.

Moreover, *Clcn7*^*Flox/Flox*^ mice were shorter than heterozygous and wild-type littermates (Fig 8A) and showed no tooth eruption when compared with wild-type littermates (Fig 8B). Both features are clinical signs of autosomal recessive osteopetrosis.

**Fig 9:**
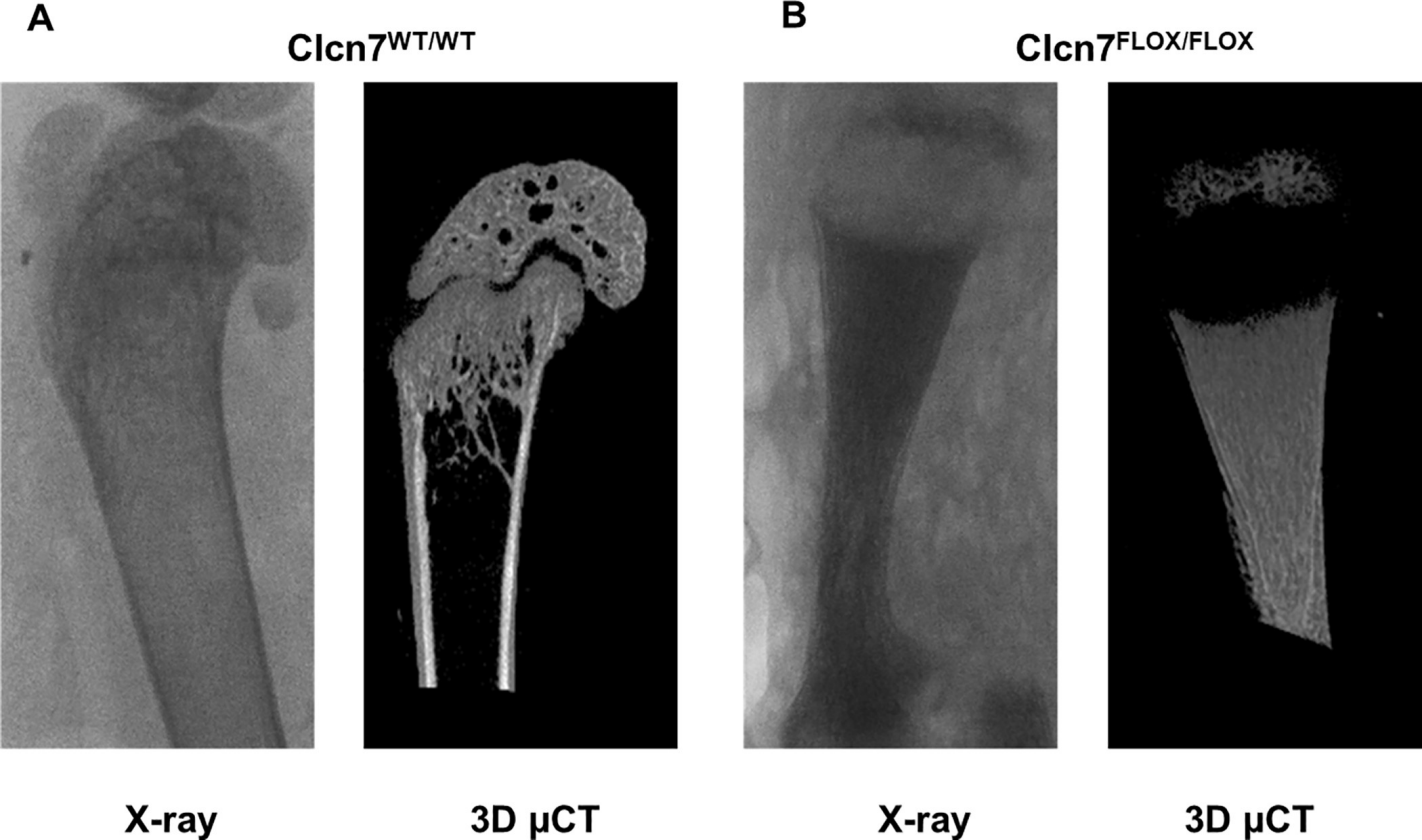
Bone phenotype of the *Clcn7*^Flox/Flox^ mice. Twenty-one day old (A) *Clcn7*^WT/WT^ and (B) *Clcn7*^Flox/Flox^ mice were sacrificed and then X-ray and μCT imaging were performed on femurs. A severe osteopetrotic phenotype with high bone mass is present in mutant mice. Pictures are representative of 5 mice per group.

After gross evaluation, femoral bones were analyzed by X-ray and μCT that revealed the 3D architecture of the bones and confirmed a severe osteopetrotic phenotype in *Clcn7*^*Flox/Flox*^ mice (Fig 9), with an extremely high bone volume/tissue volume (72 ± 4.3% in *Clcn7*^*Flox/Flox*^ and 13 ± 3.2% in wild-type littermates).

Altogether, these results revealed that the *Clcn7*^*Flox/Flox*^ mouse did not show the expected phenotype, but rather recapitulated the loss-of-function model suggesting that the genomic modification resulted in a knock-out allele.

**Fig 10:**
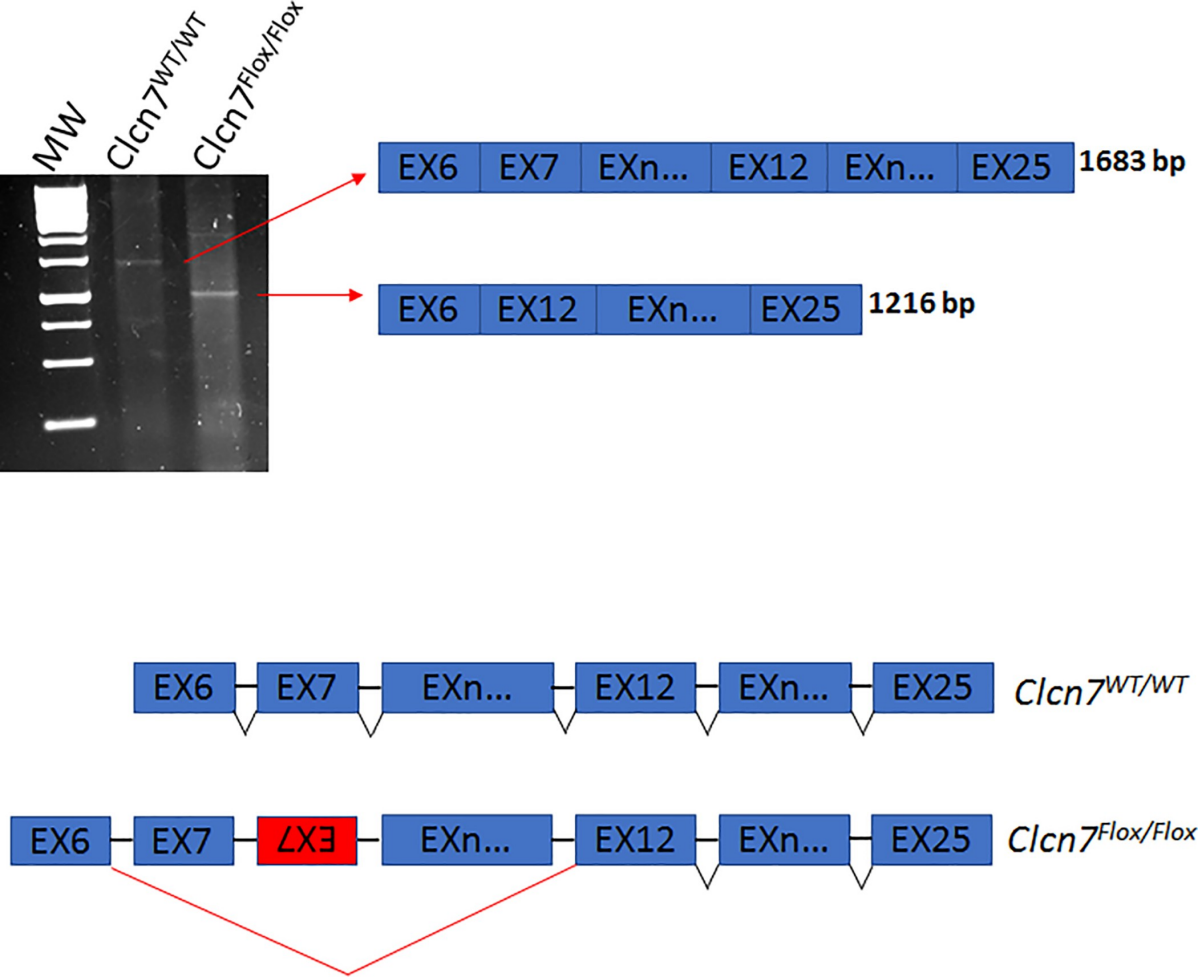
Alternative splicing analysis of *Clcn7* mRNA. (A) RT-PCR using primers that amplify a region spanning exon 6 to exon 25 was performed from bone total RNA of wild-type (*Clcn7*^*WT/WT*^) and *Clcn7*^*Flox/Flox*^ mice and analysed by 1.5% agarose gel. In wild-type animals one band, 1683 bp long, corresponding to the correctly spliced *Clcn7* transcript including 20 coding exons is present. This band is not detected in mutant mice, conversely a 1216 bp long band lacking exon 7–11 is observed.

We checked whether the modified *Clcn7* locus impairs correct RNA splicing as observed in mutant *Impad1*. Thus, we considered potential alternative splicing of *Clcn7* exons by RT-PCR using primers Clcn7Ex6 and Clcn7Ex25 spanning the coding sequence from exon 6 to 25 (Fig 10). In wild-type animals one band, 1683 bp long, corresponding to a unique transcript including 20 coding exons was present; conversely a band 1216 bp long, lacking exon 7–11 was observed in *Clcn7*^*Flox/Flox*^ mice (Fig 10). This should result in an in-frame deletion of 129 amino acid residues.

## Discussion

We have tested a Cre-mediated genetic switch combining the ability of Cre recombinase to invert a DNA fragment, depending upon the orientation of the flanking *loxP* sites, and the use of wild-type and mutant *loxP* sites in order to make recombination irreversible. The notion was that Cre recombinase-mediated inversion would place the exon bearing the missense mutation into a position where it would be spliced properly, while the inverted wild-type exon would be spliced out.

We have used this strategy to generate two conditional knock-in mice for the *Impad1* and *Clcn7* genes. Mutations in these genes cause in humans two different skeletal disorders: chondrodysplasia with joint dislocations gPAPP type and autosomal dominant or recessive osteopetrosis, respectively. *Impad1* encodes for a Golgi resident adenosine 3’,5’-bisphosphate phosphatase crucial for macromolecular sulfation [19, 24], while the *Clcn7* gene encodes for the ClC7 protein, a proton/chloride antiporter important in lysosomal acidification [26]. In bone, the ClC7 is expressed especially by osteoclasts and it has a pivotal role in bone resorption, charge-balancing the acidified osteoclast resorption lacuna. The missense mutation knocked-in the murine Impad1 was the p.Asp175>Asn corresponding to the p.Asp177>Asn detected previously in a patient [17]. The mutation knocked-in the murine Clc7 was the p.Gly213>Arg described previously in a patient with ADO2 [27, 28].

Both mouse models generated through this strategy resulted in an unexpected severe skeletal phenotype. Heterozygous floxed *Impad1* (*Impad1*^*Flox/WT*^) mice were mated to a Cre deleter mouse strain in order to generate *Impad1*^*D175N/WT*^ animals. *Impad1*^*D175N/D175N*^ mice died at birth with severe hypoplasia of the skeleton and at the biochemical level cartilage proteoglycans were dramatically undersulfated. The phenotype overlapped with the lethal phenotype described previously in *Impad1* knock-out mice generated by a gene trap approach [19, 24]. Expression studies by qPCR and RT-PCR demonstrated that the mutant *Impad1* mRNA underwent abnormal splicing with loss of exon 2 or exons 2 and 3; no mutant full length mRNA spanning exons 1–5 as in normal *Impad1* mRNA was detected. This may result in the activation of non-sense mediated decay or anyway in the synthesis of transcripts encoding for a non-functional enzyme since the active site of the phosphatase is encoded by exon 2.

A skeletal phenotype was observed also in homozygous *Clcn7*^*Floxed/Floxed*^ mice before mating to Cre deleter mice, when the wild-type exon 7 was into a position where it would be spliced properly and thus a wild-type *Clcn7* mRNA would have been generated. The skeletal phenotype observed in these mice overlaps with the *Clcn7* knock-out reported previously [25]; in fact also in this murine model abnormal splicing causing exons skipping occurred, generating a knock-out allele, likely because of the wild-type and mutant exon 7 in opposite orientation in the mutant *Clcn7*.

Thus in both animal models the skeletal phenotype parallels the phenotypes of their knock-out mice suggesting that the skeletal defects were not due to the missense mutations, but to aberrant splicing causing the synthesis of non-functional proteins. It is tempting to speculate that the two exons in opposite orientation generate a hairpin loop leading to exon skipping.

A similar strategy was devised previously in order to generate a conditional knock-in of the cAMP response element binding protein (CBP) [14]. To achieve the Cre recombinase-mediated mutation CBP^Tyr658Ala^, a targeting construct containing the wild-type exon 5 of the CBP and a mutated exon 5 (p.Tyr658>Ala) in an inverted orientation was generated. These sequences were flanked by two mutated *loxP* sites, which were positioned in a head-to-head orientation. The authors demonstrated that in ES cells the genetic switch worked properly when cells were transfected with a plasmid expressing Cre-recombinase, but unfortunately, no data were available regarding the expression at the mRNA or protein level. The generation and further characterization of transgenic animals from these ES cells has never been reported to date.

In conclusion, the Cre mediated genetic switch strategy has paved the way to the engineering of sophisticated genetic modifications including conditional point mutations, conditional rescue, conditional gene replacement and recombinase mediated cassette exchange which have been used successfully *in vitro*. However, it is worth noting that modifying the genome of eukaryotic cells to generate Cre mediated switch alleles may have some drawbacks. The repetition of endogenous splicing sites in the antisense orientation may also induce the occurrence of aberrantly spliced mRNA. In any case, it is probably safe to reduce DNA repetitions as much as possible and to test for functionality in transiently transfected cells when possible, or in ES cell clones before further proceeding to blastocyst injections.

## Supporting information

S1 Table PCR primers used in this study. (DOCX)

S1 Fig X-rays of the Impad1Flox/Flox mouse. The newborn mutant shows severe underdevelopment of the skeleton compared to the wild-type mouse.
(DOCX)
