# Supplementary material for: Testing the Cre-mediated genetic switch for the generation of conditional knock-in mice
Source: PLoS One. 2019 Mar 13;14(3):e0213660. doi: 10.1371/journal.pone.0213660 (PMC6415906; doi:10.1371/journal.pone.0213660)
Supplement: S1 Table — (DOCX) [file pone.0213660.s001.docx]

**S1 Table. PCR primers used in this study.**

Neo3-for: 5’-CAAGCTCTTCAGCAATATCACGGG-3’

Neo4-rev: 5’-CCTGTCCGGTGCCCTGAATGAACT-3’

SY08.19 5’-GCAAATAAGCAGTTACAGAATATG-3’

LRPCRneo1: 5’-AATGGGCTGACCGCTTCCTCGTGCTTT-3’

SY08.20: 5’-TGGCCTGAGACACATTCC-3’

SY08.21: 5’-CTGCTGGTACCTCCTTAG-3’

IMP3: 5’-GTTAGATTAGAAAGTACTTGAAG-3’

IMP8: 5’-CTAGTCTGCTTGTCTGGAAAC-3’

IMP7: 5’-CGAGAGCAATGTCCTTCACG-3’

IMP12: 5’-TTTTAAGATGGCATTGCCAGC-3’

SY03.9: 5’-AGGGCTCACCTGATTCGAAAC-3’

LRPCRneo1: 5’-AATGGGCTGACCGCTTCCTCGTGCTTT-3’

SY03.11: 5’-CTCTTGGCCACCCAATTC-3’

SY03.12: 5’-GGACAGAGCCTCTACAAC-3’

Clcn7Ex6: 5’-CTTCCTGGTCACCAGCCCGA-3’

Clcn7Ex25: 5’-TGTGATCGTGGCCTTCATAG-3’
