# Supplementary figures and images for: Testing the Cre-mediated genetic switch for the generation of conditional knock-in mice
Source: PLoS One. 2019 Mar 13;14(3):e0213660. doi: 10.1371/journal.pone.0213660 (PMC6415906; doi:10.1371/journal.pone.0213660)

**S1 Fig. X-rays of the *Impad1Flox/Flox* mouse**


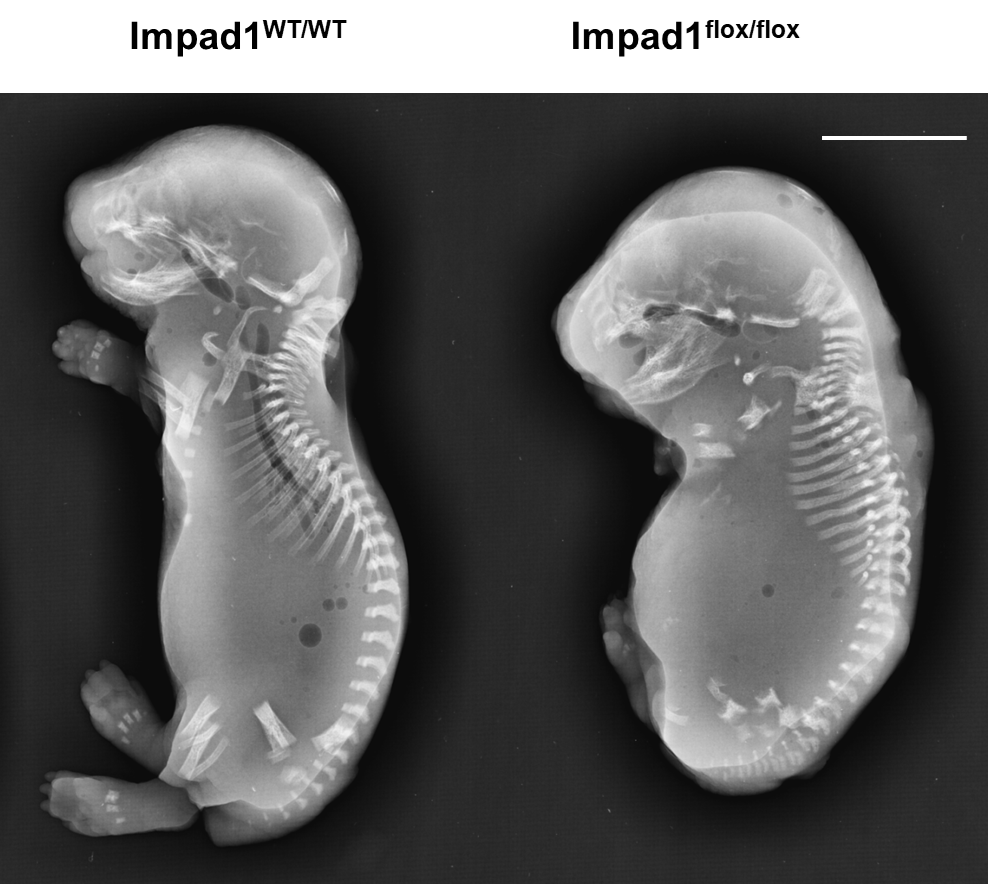

Supplement: S1 Fig — The newborn mutant shows severe underdevelopment of the skeleton compared to the wild-type mouse. (DOCX) [file pone.0213660.s002.docx]
